# Long-term health sequelae and quality of life at least 6 months after infection with SARS-CoV-2: design and rationale of the COVIDOM-study as part of the NAPKON population-based cohort platform (POP)

**DOI:** 10.1007/s15010-021-01707-5

**Published:** 2021-10-12

**Authors:** A. Horn, L. Krist, W. Lieb, F. A. Montellano, M. Kohls, K. Haas, G. Gelbrich, S. J. Bolay-Gehrig, C. Morbach, J. P. Reese, S. Störk, J. Fricke, T. Zoller, S. Schmidt, P. Triller, L. Kretzler, M. Rönnefarth, C. Von Kalle, S. N. Willich, F. Kurth, F. Steinbeis, M. Witzenrath, T. Bahmer, A. Hermes, M. Krawczak, L. Reinke, C. Maetzler, J. Franzenburg, J. Enderle, A. Flinspach, J. Vehreschild, M. Schons, T. Illig, G. Anton, K. Ungethüm, B. C. Finkenberg, M. T. Gehrig, N. Savaskan, P. U. Heuschmann, T. Keil, S. Schreiber

**Affiliations:** 1 Institute of Clinical Epidemiology and Biometry, University of Würzburg, Julius Maximilian University of Würzburg, Wurzburg, Germany; 2 Institute of Social Medicine, Epidemiology and Health Economics, Charité – Universitätsmedizin Berlin, Berlin, Germany; 3 Institute of Epidemiology, University Hospital Schleswig-Holstein, Campus Kiel, Kiel, Germany; 4 Comprehensive Heart Failure Center, University and University Hospital Würzburg, Wurzburg, Germany; 5 Division of Cardiology, Department of Medicine I, University Hospital Würzburg, Wurzburg, Germany; 6 Department of Infectious Diseases and Respiratory Medicine, Charité – Universitätsmedizin Berlin, Berlin, Germany; 7 Clinical Trial Unit, Clinical Study Center, Berlin Institute of Health, Charité – Universitätsmedizin Berlin, Berlin, Germany; 8 Department of Tropical Medicine, Department of Medicine I, University Medical Centre Hamburg-Eppendorf, Bernhard Nocht Institute for Tropical Medicine, Hamburg, Germany; 9 German Center for Lung Research (DZL), Berlin, Germany; 10 Swiss Tropical and Public Health Institute, Basel, Switzerland; 11 Department of Internal Medicine I, University Hospital Schleswig-Holstein, Campus Kiel, Arnold-Heller-Straße 3, 24105 Kiel, Germany; 12 German Centre for Lung Research (DZL), Airways Research Center North (ARCN), Kiel, Germany; 13 Institute of Medical Informatics and Statistics, University Hospital Schleswig-Holstein, Campus Kiel, Kiel, Germany; 14 Department of Neurology, University Hospital Schleswig-Holstein, Campus Kiel, Kiel, Germany; 15 Institute for Laboratory Medicine, University Hospital Schleswig-Holstein, Campus Kiel, Kiel, Germany; 16 Institute for Clinical Chemistry and Institute for Clinical Molecular Biology, University Hospital Schleswig-Holstein, Campus Kiel, Kiel, Germany; 17 Medical Department 2, Hematology/Oncology and Infectious Diseases, University Hospital of Frankfurt, Frankfurt, Germany; 18 German Centre for Infection Research (DZIF), Partner Site Bonn-Cologne, Cologne, Germany; 19 Department I for Internal Medicine, Faculty of Medicine, University Hospital Cologne, University of Cologne, Cologne, Germany; 20 Hannover Medical School, Hannover Unified Biobank, Hannover, Germany; 21 Institute of Epidemiology, Helmholtz Center Munich, Munich, Germany; 22 Department of Public Health, District Office Würzburg, Wurzburg, Germany; 23 Department of Public Health, District Office Schweinfurt, Schweinfurt, Germany; 24 Department of Public Health, District Office Berlin-Neukölln, Berlin, Germany; 25 Clinical Trial Center Würzburg, University Hospital Würzburg, Wurzburg, Germany; 26 Department of Neurology, University Hospital Würzburg, Wurzburg, Germany; 27 State Institute of Health, Bavarian Health and Food Safety Authority, Bad Kissingen, Germany; 28 German Centre for Infection Research (DZIF), Partner Site Munich, Munich, Germany

**Keywords:** Long COVID, Sars-CoV-2, On-site examination, Internal medicine, Neurological, Population-based

## Abstract

**Purpose:**

Over the course of COVID-19 pandemic, evidence has accumulated that SARS-CoV-2 infections may affect multiple organs and have serious clinical sequelae, but on-site clinical examinations with non-hospitalized samples are rare. We, therefore, aimed to systematically assess the long-term health status of samples of hospitalized and non-hospitalized SARS-CoV-2 infected individuals from three regions in Germany.

**Methods:**

The present paper describes the COVIDOM-study within the population-based cohort platform (POP) which has been established under the auspices of the NAPKON infrastructure (German National Pandemic Cohort Network) of the national Network University Medicine (NUM). Comprehensive health assessments among SARS-CoV-2 infected individuals are conducted at least 6 months after the acute infection at the study sites Kiel, Würzburg and Berlin. Potential participants were identified and contacted via the local public health authorities, irrespective of the severity of the initial infection. A harmonized examination protocol has been implemented, consisting of detailed assessments of medical history, physical examinations, and the collection of multiple biosamples (e.g., serum, plasma, saliva, urine) for future analyses. In addition, patient-reported perception of the impact of local pandemic-related measures and infection on quality-of-life are obtained.

**Results:**

As of July 2021, in total 6813 individuals infected in 2020 have been invited into the COVIDOM-study. Of these, about 36% wished to participate and 1295 have already been examined at least once.

**Conclusion:**

NAPKON-POP COVIDOM-study complements other Long COVID studies assessing the long-term consequences of an infection with SARS-CoV-2 by providing detailed health data of population-based samples, including individuals with various degrees of disease severity.

**Trial registration:**

Registered at the German registry for clinical studies (DRKS00023742).

## Introduction

Corona Virus Disease 2019 (COVID-19) is a viral disease caused by Severe Acute Respiratory Syndrome Coronavirus type 2 (SARS-CoV-2). It mainly affects the lungs, but the virus can also damage other organs in hitherto unknown ways, thereby causing long-lasting symptoms and chronic sequelae [1–3]. Globally, 142,557,268 cases of COVID-19 have been confirmed by the WHO until 22 April 2021, including 3,037,398 deaths [4]. About 8.4% of cases have been hospitalized and the mortality rate, which is consistently higher in men than in women, increases sharply with age beyond 80 years. The incidence of long-term damage and its predisposing factors are not yet fully understood and are hence subject of ongoing research [5, 6]. With a literature search in Web of Science and hand search for reviews that were conducted systematically on Long COVID or Post Covid Syndrome in the adult population, we identified nine reviews, seven of which fit our defined criteria (scoping or systematic review or meta-analysis) [6–12]. We identified one meta-analysis, four systematic reviews, one preprint meta-analysis, one preprint systematic review and one scoping review. They present studies with a mixed study population as well as studies with populations limited to only hospitalized or non-hospitalized samples. Some studies report their selected sample regarding specific diseases severities. We identified 24 cross-sectional studies or retro-/prospective cohorts reported by the reviews, who selected a mixed population (hospitalized and non-hospitalized or all severity stages) [13–31], whereas only one study reports a sample selected from the general population [32]. Approximately 60% of the studies use online questionnaires or symptom reporting as outcome, whereas for 40% of the studies, more detailed examinations including lung function, CT or laboratory measurements are reported. Data collection at follow-up ranges from 30 to 122 days post diagnosis, post onset of symptoms or post discharge. The reviews generally report high risk of bias and heterogeneity of studies as well as unclear definition of Long COVID. Regardless of those restrictions, the reviews provide an overview of the different approaches studying long-term effects of COVID-19. Across different studies, > 75% of infected participants reported persisting symptoms 2–6 months after symptom onset [16, 33, 34]. While fatigue is among the most prevalent sequelae 1–6 months after infection [6, 8–11, 16, 33–35], other consequences include headache, pain, cognitive disorders, hair loss, dyspnoea and ageusia [6, 8, 9, 11, 34, 35].

Sampling methods have differed widely between the above-mentioned studies, ranging from follow-up examination of initially hospitalized patients, via participant recruitment through social media, to the recruitment of patients who were initially treated in primary care facilities [6, 33–35]. Data related to individual symptoms, course of disease and sequelae mainly derive from hospitalized patients or patients treated in primary care. At the same time, however, data on long-term sequelae in unselected SARS-CoV-2 patients, comprising both asymptomatic and hospitalized patients, are still scarce.

Consequently, the NAPKON-POP COVIDOM-study platform has been initiated in Germany to facilitate non-interventional population-based studies with a threefold goal as follows: (1) to determine the frequency of sequelae and late complications in the general population, at least 6 months after SARS-CoV-2 infection, involving physical examination, analysis of biomaterial, functional and imaging techniques; (2) to assess and quantify the burden of disease after SARS-CoV-2 infection and the subjective impact of the pandemic on personal quality-of-life; (3) to identify determinants of the post-acute disease phase of hospitalized and non-hospitalized individuals with a SARS-CoV-2 infection, thereby contributing to an improved prevention and, if necessary, treatment of secondary diseases.

The aim of the present article is to describe the study protocol of the NAPKON-POP COVIDOM-study.

## Methods

### Background and basic study design

**Fig. 1:**
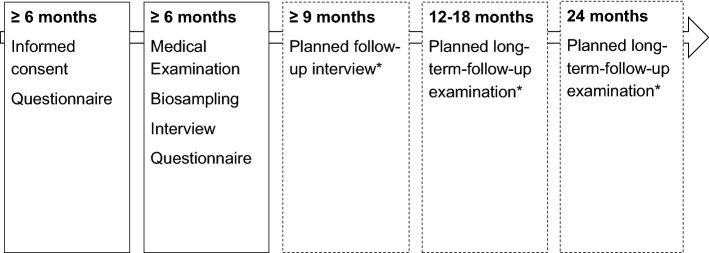
Schematic study design in relation to individual time after infection. *Dependent on subsequent funding

In response to the SARS-CoV-2 pandemic, a nationwide network of German university hospitals has been established to coordinate SARS-CoV-2-related research, the Network University Medicine for COVID-19 Research (NUM). Under the auspices of the network, three cohorts of SARS-CoV-2-infected or COVID-19-affected patients were established as the National Pandemic Cohort Network (“Nationales Pandemie Kohorten Netz”, NAPKON) to coordinate clinical epidemiological cohort activities. The three cohorts have different sampling strategies as well as different inclusion and exclusion criteria (hospitalized: NAPKON-HAP, cross-sectoral: NAPKON-SÜP, population-based: NAPKON-POP). The main purpose of the NAPKON-POP COVIDOM cohort is to investigate long-term organ damage and morbidity, starting at least 6 months after SARS-CoV-2 infection, using a population-based approach. COVIDOM includes participants at all levels of disease severity, from asymptomatic to very severe. The platform comprises three recruitment sites covering three different target areas, namely Kiel (Schleswig–Holstein, most northern federal state of Germany), Würzburg (Lower Franconia, located in the south-east of Germany and belonging to the federal state of Bavaria) and the Neukölln district of Berlin (capital of Germany). Individuals with a history of polymerase chain reaction (PCR)-proven SARS-CoV-2 infection and residing in one of the target regions are invited to participate (see below for details). As all proofed infected individuals are registered at their local health authority (“Gesundheitsamt”) in Germany irrespective of the location of the PCR test, the study sites collaborated with the local health authorities of their region in order to invite all individuals infected during a certain time span (to be able to do examinations at least 6 months after infection). The current assessment of participants includes a comprehensive interview and a detailed clinical examination (Fig. 1). Follow-up examinations after 1 and 2 years are envisioned. The study is registered at the German registry for clinical studies (DRKS00023742) and at ClinicalTrials.gov (NCT04679584).

### Inclusion and exclusion criteria

The NAPKON-POP COVIDOM-study inclusion criteria comprise (i) a positive PCR test for SARS-CoV-2 (positive pathogen testing) at least 6 months before inclusion in the study, (ii) primary residence in one of the three study regions, (iii) age ≥ 18 years at the time of recruitment (Berlin) or infection (Kiel, Würzburg) and (iv) written informed consent to participate in the study. An acute re-infection with SARS-CoV-2 at the time of the interview or at the scheduled site visit represents an exclusion criterion.

### Sampling

Sample size calculation is based upon the likely prevalence of outcomes and on the respective probability of seeing such cases during study examination. In total, at least *n* = 1320 participants are expected to be recruited at the three sites (Kiel: *n* > 700, Würzburg: *n* = 300, Berlin: *n* = 300). Sample sizes differ between the three regions due to different capacities of the local study sites. At an individual site (*n* = 300), there is a 95% chance of observing at least once a sequela that occurs with 1% frequency in the population. In the total cohort (*n* = 1320), there would be a 99% chance of observing at least six cases. With percentile ranks of a quantitative outcome 5% above or below the reference in another cohort, the power to detect this difference at a 2-sided significance level of 5% would already be > 80% if *n* = 300.

### Recruitment strategies

The recruitment strategies differ slightly between the three study sites, focusing on different social and epidemiological aspects in order to achieve an optimal population representation, but all pursue a population-based approach and conduct similar interviews and clinical examinations. In order to recruit an unbiased sample of the source population irrespective of age, sex or hospitalization, all infected individuals in the study regions were invited via the health authorities. Therefore, the invitation reached everyone equally regardless of initial disease severity or media use competence. Participants received an incentive for participating, so even individuals without impairments (who might not expect any health benefits) had an interest to participate.

Schleswig–Holstein: The responsible local health authorities of Kiel and the four surrounding local districts of Plön, Rendsburg-Eckernförde, Schleswig-Flensburg and Neumünster were asked to identify individuals with proven SARS-CoV-2 infection, irrespective of the clinical course or the severity of the infection. Invitations were staggered and, depending on the sub-study region, individuals were enrolled who were infected until October 2020 or January 2021. In total, 2970 individuals in the Schleswig–Holstein study region were actively invited to participate in NAPKON-POP COVIDOM-study. To date, about 42% of invitees have responded (by July 2021). Based upon the assumption that manifestation of the long-term sequelae of a SARS-CoV-2 infection might be independent of the initial disease severity, no additional exclusion criteria were applied in Schleswig–Holstein.

Würzburg: The responsible health authority of Würzburg and the neighbour local district of Schweinfurt were asked to identify adults with proven SARS-CoV-2 infection of any level of severity who got infected between March and September 2020, and to invite them to participate. In addition to the above-mentioned exclusion criterion, residents of long-term care facilities (senior homes) were excluded for logistic reasons if possible. 2163 invitations were sent out in December 2020 and about 23% of invitees have responded (by July 2021). To increase the representativity of the invited individuals to the underlying population of all SARS-CoV-2-infected individuals in the target region, the sample was stratified based upon age (group), sex, hospitalization status and approximate time of the infection (1st vs. 2nd wave of pandemic). More specifically, age groups of 18–29, 30–45, 46–60, 61–79, 80+ years were selected in a ratio of 25:25:25:15:10; infection in pandemic phase 1 vs. phase 2 at ratios of 75:25–70:30; men and women at ratios of 40:60–60:40. The ratio of hospitalized to non-hospitalized individuals was approximately 10:90. Based on the infection rates observed in Germany, there was a peak of infection rates in late March 2020 and a slight increase again since June 2020 (start of pandemic phase 2). The second peak was observed in mid-December 2020, and the third in mid-April 2021. No information about dominating variants is available for our recruited participants.

Berlin: After the first known SARS-CoV-2 case in Berlin on March 2020, local health authorities reported more than 2000 cases in the city by the end of March 2020. Seven months after the first known case, 2099 cases were reported in Berlin Neukölln, making it the second most affected district in Berlin. The local health authority of the Neukölln district of Berlin (serving a population of 330,000) has sent invitations to the first adults who were registered as being SARS-CoV-2 positive in 2020, including all subjects with confirmed SARS-CoV-2 infection starting in March 2020 up to the day when 300 participants will have undergone the physical examination protocol. This will probably be in the same time span as in Würzburg, i.e. until September 2020. Residents of long-term care facilities (senior homes) are excluded from participation. The first 1700 adults who were registered by the health office in the district of Neukölln since March 2020 were consecutively invited to participate in COVIDOM. In the first wave, about 40%of invitees from Berlin-Neukölln have responded and expressed an interest in the study. In contrast to Schleswig–Holstein and Würzburg, Berlin-Neukölln has a high proportion of residents with a migration background, thereby complementing the population spectrum of the two other cohorts included in NAPKON-POP COVIDOM-study.

### Data collection: comprehensive interview and detailed clinical characterisation

Participants are asked to complete a comprehensive health questionnaire, administered as either a telephone interview, in paper-based form or as an online questionnaire (Table 1). Afterwards, participants are invited to an on-site examination of 4–5 h duration at the clinical-epidemiological study centre of the respective university hospital (Fig. 1). Both the initial and on-site questionnaire as well as the examination form the baseline assessment. One questionnaire is filled out at the time point of study inclusion and a more detailed questionnaire is filled out during waiting phases of the on-site examination, to lower the burden of the participants.

**Table 1:** Contents of baseline examination (≥ 6 months after infection)

	Target/domain	Instrument/device
Interdisciplinary modules		
Self-administered questionnaires	Socio-demographic/economic factors	
	Tobacco and alcohol consumption	
	Lifestyle (physical activity, nutrition)	
	Sleeping habits	PSQI
	Pandemic-related behaviour and worries	
	Depression	PHQ-8
	Anxiety	GAD-7
	Trauma, stress	ITQ, PSS, BRS
	Quality of life, health status	EQ-5D-5L
	Social network	6-ILS
	COVID-19 symptoms	
Medical interview (conducted by physician or nurse)	Medical history, including: Cardiovascular/cerebrovascular disease Metabolic disease Neurological and psychiatric disorders Musculoskeletal disorders Infectious disease Rheumatic disease Operations Medication, vaccination COVID-19 treatment	
Organ modules		
Anthropometry	Size, weight	Measuring tape, stick
	Body composition	Bioelectrical impedance scale SECA mBCA 515, BIACORPUS RX Spectral, NutriBox Fa. Data Input
	Vital signs (blood pressure and heart rate, temperature)	Omron HEM 705 IT, boso carat professional, pulox PO-300 Finter Pulsoxymeter Univerhealth infrared-thermometer, in-ear thermometer
Cardiology/angiology	Symptoms	
	Heart failure	KCCQ
	Echocardiography, including: Cardiac structure and dimensions Valve function Pump function Wall movement Relaxation dysfunction	Vivid E95, Philips Epic Cx
	Carotid intima-media-thickness*	Vivid E95
	Electrocardiogram	GE Healthcare Cardio Soft V6.73, Schiller AT-2
Chemosensorics	Olfactory and gustatory function*	Self-MOQ, QOD, SNOT-22
	Sensoric function: identification, discrimination* and threshold*	Sniffin’ sticks and taste strips tests (Burghart Messtechnik GmbH)
	Nasal endoscopy*	Karl Storz TELE
	Inspection of oral cavity*	PACK + Endoscope
Hepatology*	Liver-check-up	
	Sonography and elastography of liver	FibroScan^®^, Canon Aplio i800
Neurology	Symptoms after infection	
	Basic neurological examination (efferent and afferent nerve fibers, basal ganglia, cerebellum)*	Rydell Seiffer Tuning Fork, Coroflex Reflex Hammer, Diagnostic Lamp
	Grip strength*	Patterson Medical Jamar^®^ Plus Hand Dynamometer
	Fatigue screening	FACIT-F, MFI, PVT*
	Global cognitive screening	MoCA
Geriatrics*	Motoric function	Timed ‘up and go’ test, 6-min-walking test, trail making test A/B, hand grip force
	Function and disability	CFS, LLFDI, DIA-S
Pneumology	Dyspnoe and breathing	mMRC, MDP, ACT, CAT, PROMIS-Dyspnea
	Physical activity	GPAQ
	Lung function: Body plethysmography Spirometry Diffusion gas Oxygen saturation Airwave oscillometry FeNO	Vyaire Vyntus ONE Bodyplethysmograph including diffusing capacity unit, radiometer ABL80 Flex CO-OX, THORASYS tremoFlo C-100, Circassia NIOX VERO
Laboratory	Routine laboratory	
	Immunological, endocrinological, myocardic laboratory	
	SARS-CoV-2 antibody and PCR testing	
	Study biomaterials samples	

*FeNO* fraction of nitric oxide in exhaled breath, *PSQI* Pittsburgh sleep quality index, *PHQ-8* patient health questionnaire, *GAD-7* generalized anxiety disorder, *ITQ* international trauma questionnaire, *PSS* perceived stress scale, *BRS* brief resilience scale, *EQ-5D-5L* 5-level EuroQol five dimensions, *6-ILS* 6-item loneliness scale, *KCCQ* Kansas City cardiomyopathy questionnaire, *Self-MOQ* self-reported mini olfactory questionnaire, *QOD* questionnaire of olfactory disorders, *SNOT-22* sino-nasal outcome test, *FACIT-F* functional assessment of chronic illness therapy-fatigue, *MFI* multidimensional fatigue inventory, *MoCA* montreal cognitive assessment, *CFS* clinical frailty scale, *LLFDI* late life function and disability index, *PVT* psychomotor vigilance test, *DIA-S* depression-im-alter-skala (depression in elderly), *mMRC* modified medical research council questionnaire for assessing the severity of breathlessness, *MDP* multidimensional dyspnea profile, *ACT* asthma control test, *CAT* COPD assessment test, *PROMIS* patient-reported outcomes measurement information system, *GPAQ* global physical activity questionnaire

*Additional data element, not being collected across all sites

A pre-defined, harmonized dataset is being collected from all participants including items of the German Corona Consensus Dataset (GECCO) [36]. Prioritization of variables has allowed the collection of both core data that are comparable between study sites and supplemental data according to the capacity and scientific interests of the individual study site (Table 1). The baseline examination includes a detailed assessment of lung function, including forced spirometry, body plethysmography, diffusing capacity of the lungs for carbon monoxide (DLCO), fraction of nitric oxide in exhaled breath (FeNO) and an assessment of small airway dysfunction by impulse oscillometry. The cardiovascular domain comprises two repetitive blood pressure measurements (2 min apart), an electrocardiogram at rest and a detailed transthoracic echocardiography according to the standards of the German Centre for Cardiovascular Diseases (DZHK). These data yield comprehensive information on cardiac structure and function, including valvular structure and function. The neurological examinations include an assessment of somatosensory function, cognition and memory. In addition, detailed chemosensory assessments are performed including smell and taste tests. At the study sites Kiel and Würzburg, a sonographic analysis of liver function is performed. Anthropometric measures comprise height, weight, waist circumference and bioimpedance data. Routine laboratory measurements include blood count, electrolytes, uric acid, creatinine, albumin, and haemoglobin, among others. In addition, N-terminal pro-brain natriuretic peptide (NT-proBNP), cardiac troponin, interleukin-6, SARS-CoV-2-RT-PCR and SARS-CoV-2 IgG and IgA IA are measured and several biosamples—which are standardized throughout NAPKON—are collected, including blood and urine. These samples are stored locally at the study sites at − 80 °C or nitrogen repository for future analyses. Trained and certified personnel perform all procedures according to detailed standardized operation procedures (SOPs). Waiting times between examination modules are filled with self-administered demographic, epidemiological, psycho-social and symptom-related questionnaires.

Followed-up examinations are planned for various defined time points after baseline (Fig. 1), oriented at the baseline data but with possible adjustments based upon intermittent research findings on Long COVID.

### Primary and secondary outcomes

Primary outcomes are defined as persistent symptoms and/or functional impairment after a minimum of 6 months after infection, documented as type and frequency of symptoms and impairment. Secondary outcomes include symptoms due to chronic morbidity following SARS-CoV-2 infection, as well as quality-of-life, health status and health care utilization. Therefore, data on the persistence of symptoms, the persistence or development of organ damages, the development of disease following infection, self-rated health status, quality-of-life and the socioeconomic situation as well as out- and in-patient medical care after infection are documented.

### Data management and storage

**Fig. 2:**
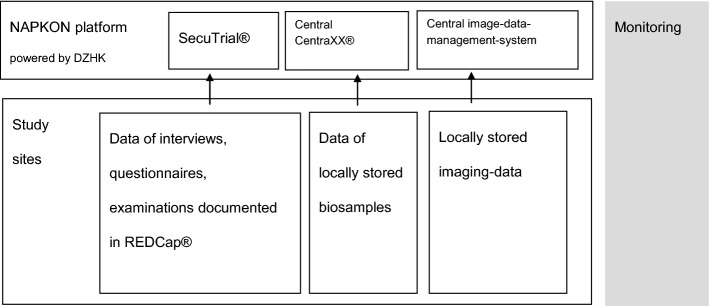
Data storage and quality assurance. *DZHK* German Centre for Cardiovascular Research, *SecuTrial®/REDCap®* EDC system, *CentraXX* electronic biobanking research portal

Personal information (e.g., name, address) and clinical data (e.g., from questionnaire and clinical examinations) are stored in strictly separated IT systems with access secured via username and password. The personal information is accessible exclusively for employees (re-) contacting participants. At all study sites, source data are collected pseudonymized in local electronic data capture systems using REDCap^®^, followed by secure transmission to a SecuTrial^®^ instance facilitating central data capturing within the NAPKON network. Data of biosamples are pseudonymized and documented in a central CentraXX^®^ instance whereas pseudonymized image data are uploaded to an electronic image data management system (BMDS). SecuTrial^®^, CentraXX^®^ and BDMS are part of the central clinical research platform of NAPKON (Fig. 2). At the Kiel study site, biosamples are stored in a − 80 °C or nitrogen biospecimen repository of the Healthcare-embedded Biobank at the UKSH, at the Würzburg study site in a − 80 °C or nitrogen biospecimen repository of the Interdisciplinary Bank of Biomaterials and Data Würzburg (ibdw) and at the Berlin study site in a − 80 °C biospecimen repository of the Charité-Universitätsmedizin Berlin (Campus Virchow).

### Quality assurance

COVIDOM personnel received intensive, standardized training. The acquisition and processing of these samples follow a strict and well-established protocol ensuring exceptionally high sample quality (see SOP https://www.bbmri.de/covid-19/nationales-pandemie-kohorten-netz/bioprobensammlung/). All acquired data in COVIDOM are quality-controlled by a dedicated quality control manager. In addition, automated plausibility checks are implemented. Quality management includes two steps of data review, possible queries and approval. Upon final approval by the quality control manager, the data are uploaded to the SecuTrial^®^ database of NAPKON, where they are centrally stored.

### Ethics

The COVIDOM study protocol and procedures were approved by the local ethics committees (Kiel: D537/ 20; Würzburg: 236/20_z; Berlin: according to the coordinating study centre Kiel). Local data protection officers have been informed as required. All participants gave written informed consent prior to their inclusion in NAPKON-POP COVIDOM-study.

### Statistical considerations

The data collected in NAPKON-POP COVIDOM-study will become available to interested research groups through the standardized use-and-access procedures of NAPKON. Exploratory analyses, in connection with defined research questions, are currently planned and will include all data available in all subgroups at the time.

### Current status of recruitment

Recruitment is still ongoing and will probably end in December 2021. As of 5 May 2021, the recruitment status at the three sites was as follows: In Schleswig Holstein, 2970 invitation letters had been sent out, 576 individuals had completed the initial online interview and 509 participants had completed their health assessment at the examination centre. In Lower Franconia (Bavaria), 2163 individuals had been invited to participate, 448 had contacted the study centre and 174 had completed the initial interview and the baseline examination. In Berlin-Neukölln, 300 individuals had been invited, 141 had contacted the study centre, 120 had filled out the online interview and 42 participants had been examined at the clinical study centre.

## Discussion

COVIDOM follows a population-based, detailed approach to study symptom persistence, potential sequelae and long-term organ damage at least 6 months after an acute SARS-CoV-2 infection. To this end, comprehensive clinical data and biosamples are collected at three sites covering different regions in Germany. The detailed study protocol guarantees consistent data quality across the three sites, thereby also ensuring comparability to other research initiatives within the frameworks of NAPKON and NUM*.*

So far, only a small number of studies focussing on the long-term consequences of SARS-CoV-2 infection have adopted a population-based approach. Population-based cohort studies on COVID-19 currently include infected, potentially infected and uninfected participants [37, 38]. To the best of our knowledge, these studies have focused primarily on the collection of information about demographics, symptom persistence, the psychological and socioeconomic consequences of the pandemic, about measures to fight the pandemic and about chronic conditions. In most cases, this information has been obtained through interviews or self-administered questionnaires. Even though there exist other systematic collections of laboratory and clinical data [39], medical examinations and a systematic collection of biomaterials often are not performed in population-based studies. Instead, medical follow-up data and biosamples are mainly collected in non-population-based studies with hospitalized participants [5, 33, 37, 38]. The NAPKON-POP COVIDOM cohort thus complements other studies on SARS-CoV-2 infection and COVID-19 by providing original population-based data on the long-term consequences of these health problems and expands other research initiatives in many ways.

First, data are collected by a population-based approach, including participants with a proven SARS-CoV-2 infection, but irrespective of the initial clinical course. As such, the NAPKON-POP COVIDOM-study also includes individuals in whom the initial infection was mild or almost asymptomatic, but who might still suffer from delayed symptoms or subclinical organ damages. Data on whether such mild initial clinical presentations still confer a relevant risk for long-term morbidity are currently lacking. Second, comprehensive (4–5 h) clinical examinations are performed in dedicated study sites, including detailed assessments of the heart, lung, neurological status, taste and smell, anthropometry, and mental and emotional health. Patients are directly examined for potential organ damages to assess morbidity and new onset of disease. In addition, epidemiological parameters, medical history and psychosocial consequences are recorded through interviews and questionnaires. Third, examinations in COVIDOM meet all relevant clinical and epidemiological standards of data acquisition. This means that all data are obtained by trained personnel and physicians in the university hospitals of Kiel, Würzburg and Berlin (Charité). Moreover, all clinical data are subjected to expert review. Regarding epidemiological standards, all data are obtained following detailed SOPs, data acquisition is supervised, and the obtained data are quality-checked by a quality control manager. Data are uploaded into a central database within NAPKON where additional quality control features have been implemented. All data can be applied for via standardized use-and-access procedures (https://proskive.napkon.de/). Finally, comprehensive on-site laboratory testing is performed and additional biosamples (blood, urine, saliva) are collected and stored for future analyses.

Although the current study design has several strengths, including population-based stratification, the involvement of different study sites and a well-standardized dataset, it also has some limitations. First, it is possible that, even though the NAPKON-POP COVIDOM-study aims at including participants at all severity levels, sicker individuals are likely to be underrepresented, as very old or very impaired individuals are less able to come to the study centre. On the other hand, however, it is also possible that sicker individuals in particular will participate because they expect health benefits from the study. Additionally, more individuals may be included who had significant symptoms during their infection because they had a higher chance of being tested at the beginning of the pandemic. In order to verify the sampling procedure, a non-responder analysis will allow us to compare participants and non-participants of the invited source population with regard to sex, age and hospitalization status. Second, the baseline assessment includes a retrospective collection of severity of symptoms and infection from the participant. Therefore, there might be the risk of a recall bias with respect to the initial disease phase, which may lead to an over- or underestimation of symptoms and clinical signs. This should be taken into account when interpreting the results. Nevertheless, the use of available doctor’s letters and information on medication from the treatment of infection will help us estimating the severity of the initial COVID disease. Third, comparison of the COVIDOM study population with comparable control groups is necessary. Comparisons are needed in order to distinguish between symptoms of Long COVID and the impact of policy measures for the population during the pandemic (e.g., depression and fatigue). They are additionally important to identify increased diagnosis of disease due to aging or more detailed screenings in the context of study examinations, which would otherwise be falsely attributed to COVID-19. Possible control cohorts with a non-COVID study population from the general population already exist at all three sites. Therefore, no control subjects were included into the COVIDOM-study. Fourth, the response rate may be limited, as no reminder of the invitation letter with contact details of the study centres was sent out by the health authorities to non-responders due to the fact that the health authorities did not know who responded and who did not. We decided against linking any personal data from the health authorities and study centre ensuring better data protection. Therefore, only the local health authorities are inviting the potential participants by contacting all registered SARS-CoV-2 infected individuals.

## Conclusion

In summary, the present manuscript provides an overview of the methodology and study design of the population-based cohort study COVIDOM as part of the NAPKON-POP. The three study sites involved recently started recruitment, in cooperation with local health offices. The study design combines epidemiologic data collection with on-site clinical investigations. In addition to the collected data, standardized biosamples are available for collaborative research, thereby promoting networking of scientific activities related to the COVID-19 pandemic. Jointly, research using the NAPKON-POP COVIDOM-study and other population-based resources will help to better understand the long-term consequences of SARS-CoV-2 infection and to develop effective means of prevention and treatment.

## Data Availability

Data are accessible for collaborative research upon request via a transfer point of NAPKON (more information is available on the NAPKON website https://napkon.de/; https://proskive.napkon.de/).
